# Influence of the moisture and ash content in flue gases on the performance of adsorption processes using activated carbons to capture the CO_2_ for reuse in greenhouses

**DOI:** 10.1016/j.heliyon.2024.e40346

**Published:** 2024-11-16

**Authors:** R. López Pastor, M.G. Pinna-Hernández, J.A. Sánchez Molina, F.G. Acién Fernández

**Affiliations:** a Department of Chemical Engineering, University of Almería, Carretera de Sacramento s/n 04120 La Cañada de San Urbano, Almería, Spain; b Department of Informatics, University of Almería, Carretera de Sacramento s/n, 04120, La Cañada de San Urbano, Almería, Spain; c Solar Energy Research Centre (CIESOL), Joint Centre University of Almería-CIEMAT, Almería, 04120, Spain

**Keywords:** Global warming, Modelling, Physical adsorption, Economic analysis, Humidity

## Abstract

This work studies the influence of flue gas composition, its moisture and ash content, on the efficiency of a CO_2_ adsorption/desorption process to capture the CO_2_ from flue gases along with its subsequent reuse in greenhouse CO_2_ enrichment (Patent ES2514090). The influence of the inlet flow rate, moisture, and ash content were analysed. The experimental conditions were based on those that are achievable under real operating conditions, namely an inlet flow rate from 1.2 to 4.8 L per minute, humidity from 3 % to 65 %, and an ash concentration from 0 % to 1 %. The results show that the inlet flow had no effect on the adsorption capacity but that there was a reduction in the adsorption capacity at the higher humidity and ash content levels studied, of 10.5 % and 21 %, respectively. The data were used to develop models based on the Langmuir and Freundlich isotherm that fitted the experimental data with a reliability of 100 % and 80.1 %, respectively. This model was used to optimize the combustion gas variables and thus their influence on the final CO_2_ adsorption/desorption capacity. The techno-economic analysis performed confirmed a total cost reduction of 12 % when using the optimal combustion gas conditions (a relative humidity of 3 % and an ash concentration of 0 %) versus the worst gas conditions (a relative humidity of 65 % and an ash concentration of 1 %), which resulted in a saving of 60 % by avoiding the use of liquified CO_2_. These results confirm the technical and economic viability of the proposed technology and its potential contribution to improving the environmental and economic sustainability of agricultural food production.

## 1 Introduction

Nowadays one of the biggest environmental issues facing the planet is global warming, such as the Kyoto protocol, in which they commit to reduce greenhouse gas (GHG) emissions around 5.2 % lower than the 1990 level. Likewise, the Copenhagen Accord requires the increase in global warming will not be over 2 °C above the preindustrial CO_2_ concentration by 2100. Among the GHGs we find CO_2_, methane, and chlorofluorocarbons. In fact, CO_2_ is the main anthropogenic GHG in the atmosphere because it contributes to more than 60 % of global warming due to the huge amount of it being emitted. To date, preindustrial CO_2_ concentration was around 300 ppm in the atmosphere, but now CO_2_ concentration in the atmosphere has increased to over 400 ppm. Furthermore, CO_2_ concentration is expected to continue increasing to 550 ppm by 2050 which is inevitable even if CO_2_ emissions remain stable. The increase in world population and therefore energy demand, which is expected to increase by around 53 % by 2030, makes it impossible to immediately halt CO_2_ emissions [[1], [2], [3], [4], [5]]. The increase in population is also associated with an increase in the demand for food production. Currently, modern agriculture is responsible for providing food for 6 billion people. However, agriculture has negative impacts on the environment such as GHG emissions, nutrient and toxin contamination in groundwaters and surface waters, and the loss of ecosystem services, etc. Moreover, the food demand is expected to be highly increase in the future because by 2050, the human population will reach approximately 9 billion people. It is necessary to increase food production yield and sustainability [[6], [7], [8], [9], [10]].

On the other hand, CO_2_ has been the subject of concerted research efforts to understand the effect of this molecule on crop growth in different climatic regions. From these studies, it has been concluded that higher CO_2_ concentrations than the atmospheric value enhance the photosynthesis rate, a volume fraction of 0.035–0.038 %, to a volume fraction of 0.1 %. This has attracted interest regarding CO_2_ enrichment in greenhouses over recent years as it is an important aspect when considering how to achieve optimal growing conditions, which in turn affect both production yield and economic viability [11,12]. Employing CO_2_ enrichment in greenhouses enables food production to be increased up to 25 % and provides an alternative use for the CO_2_ recovered from combustion gases [[12], [13], [14], [15], [16]]. Traditionally, CO_2_ enrichment is carried out by direct injection of pure CO_2_ from liquefied CO_2_, but one can also use the CO_2_ generated from biomass boilers [17].

Much of the research has worked on developing methods to mitigate CO_2_ emissions and improve food production using a range of strategies. The capture of CO_2_ and its use in food-related processes has been widely studied, but CO_2_ capture requires a large amount of energy, around 3.9 MJ/kg CO_2_ [18]. Greenhouses need to maintain a stable temperature despite the temperature variation outside; this is achieved by artificial heating that can be provided by introducing hot air inside the greenhouse (air heaters) or by circulating hot water through ducts in the floor (radiating floors). The air or water can be heated by a biomass boiler and then the combustion gases, which are rich in CO_2_, can be used for the direct production of crops inside the greenhouse. Specifically, the CO_2_ can be captured from combustion gases and stored with activated carbon (AC), then used for CO_2_ enrichment, which improves the sustainability of the process [13,19]. The reuse of CO_2_ is not only limited to greenhouses, the captured CO_2_ can be used to produce methane and other hydrocarbons [20], or even be used to meet CO_2_ demand itself [[21], [22], [23]].

The patent, referenced as the “Combined system of heating and carbon enrichment from biomass” (ES2514090), proposes heating the greenhouse and capturing the CO_2_ produced with activated carbon to later use it for CO_2_ enrichment to improve the sustainability of both processes [24]. A biomass-based chamber heating system raises a greenhouse's temperature when necessary, normally at night, with the CO_2_ produced being stored in an AC bed by means of an adsorption-desorption system. Then, when the greenhouse's CO_2_ concentration drops below the optimal range of 0.1 % volume fraction, the captured CO_2_ is released into the greenhouse until the desired value is reached (based on the crop) [13,24].

A previous study demonstrated that improvements in CO₂ adsorption capacity could be achieved by modifying the operating variables in the adsorption process [25]. The aim of this paper is extend the study of operation variables through to optimize the combustion gas variables in the adsorption process (the inlet flow, the inlet gas water vapor content, and the ash concentration), which is carried out using a combined prototype system to improve CO_2_ captured per kg of AC and the profitability of the process. For this purpose, the CO_2_ adsorption capacity of commercial activated carbon was evaluated as a function of the combustion gas variables. Moreover, the effect of these variables was then modelized to predict their effect on the adsorption capacity of the AC. Finally, an economic study was performed. Additionally, the negative effects and possible degradation was evaluated that might occur in the adsorbent material (the active carbon) as a result of poor conservation.

## 2 Materials and methods

### 2.1 Experimentation

**Fig. 1:**
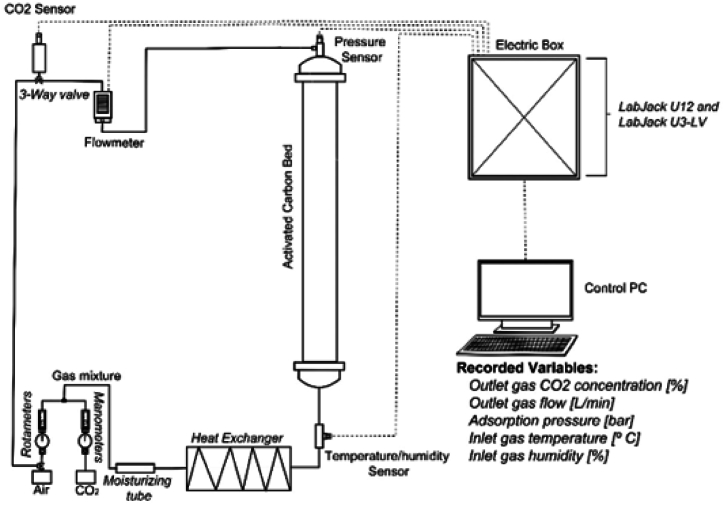
Experimental adsorption system.

The experimental adsorption system was designed to carry out adsorption-desorption tests over different adsorbent materials (Fig. 1) in previous works, considering different variables (temperature, pressure, CO_2_ concentration, moisture, and ash, etc.). To obtain the gas mixture at the desired CO_2_ concentration, gas from a CO_2_ gas cylinder is mixed with ambient air provided by a compressor. The flow and pressure for both gases are manually regulated using a rotameter and a manometer, respectively. The gas mixture passes by a moisturizing tube and a heat exchanger where the gas humidity and temperature are modified; then, a temperature/humidity sensor records these data prior to the mixture arriving at the adsorption bed. Activated carbon bed has a volume of 10 L, into which 2 kg of activated carbon is introduced, thus up to 95 % of the column being occupied by the adsorbent. The estimated porosity of the material is 0.3, thus the volume of the column occupied by the solid phase corresponds to 7.0 L. The adsorption bed is made from polyvinyl chloride (PVC) and methacrylate, and has a pressure sensor. The gas mixture crosses over the adsorption bed, putting it in contact with the adsorbent material inside, and then exits the system passing by a rotameter, a flowmeter, and a CO_2_ sensor. Moreover, the system has a cleaning channel to clean the outlet, eliminating the accumulated CO_2;_ this avoids recording errors at the CO_2_ sensor. The information recorded by the sensors is received by a data acquisition board (LabJack U3-LV, Azotech, USA) and transferred to the control PC where the data are supervised and registered using the DAQFactory program.

The adsorbent material used was “B-PURE 10-NB” AC from DESOTEC [26]. Activated carbon was selected instead of zeolites or Metal Organic Framework (MOFs) mainly because of the low cost of these materials and their low moisture sensitivity, which makes them a good option for treating gases that have a significant humidi6y content, such as biomass combustion gases [2,4,[27], [28], [29]]. The characteristics of the “B-PURE 10-NB” AC are shown in Table 1 [26]. In this study, no other adsorbent material was used in the tests due to what was previously mentioned, since the combustion gas is expected to present humidity around 10.2–15.2 % [24]. Zeolites and MOFs are materials with high adsorbent capacities, but they saturate quickly in the presence of humidity, which makes them a non-recommended material if the process is going to work directly with wet gases [[30], [31], [32], [33]]. A recovery studied of CO_2_ obtained a drop from 78.5 % to 60 % when moving from dry to wet flue gas while the productivity dropped by 22 % [30]. Depending on the base material of which they are composed, MOFs can even lose their adsorbent capacity after thermal regeneration of the material. What's more, after exposure to 70 % RH and subsequent thermal regeneration, only about 16 % of the initial CO_2_ adsorbance capacity was recovered from the Mg/DOBDC MOF [33].

**Table 1:** Activated carbon “B-PURE 10-N” characteristics [26].

Variable	Value	Units
Original material	Nutshell	
Particle size	2.36 - 4.75	mm
Iodine index (Min.)	1050	mg/g
Apparent density	480	kg/m^3^
CTC^a^ value	62	%
BET surface area	1110	m^2^/g
Hardness	97	%

a CTC: Carbon Tetrachloride Activity.

### 2.2 Methodology

The adsorption-desorption capacity of the AC was estimated for each test employing (1), (2), where CO_2_ Ads is the CO_2_ adsorbed [g CO_2_/kg AC], CO_2_ Des is the CO_2_ desorbed [g CO_2_/kg AC], Q is the outlet gas flow [L/min], P is the adsorption pressure [P], Ci is the gas mixture's CO_2_ concentration [%], Ct is the CO_2_ concentration in the outlet gas [%], m_AC_ is the activated carbon in the bed [kg] and p_CO2_ is the CO_2_ density [g CO_2_/L]. The mass of CO_2_ retained in the activated carbon bed that is not filled with activated carbon is subtracted from the value using the K_loss_ value, which is calculated by considering equation (1.5), where *V* represents the liters of the empty activated carbon bed [L] and considering a molar mass about 44.01 g/mol for CO_2_ molecules.

(1) $${CO}_{2}\;Ads=\left[\left(Q\times P\times \left(\left(C_{i}-C_{t}\right)/100\right)\times \left(10\;\left[s\right]/60[s/\min]\right)\times p_{{CO}_{2}}\right)-K_{loss}\right]/m_{AC}$$

(1.5) $$K_{loss}=\frac{P\left[atm\right]\times V\left[L\right]}{R\left[L\;atm/mol\;K\right]\times T\left[K\right]\;}\times 44.01\;\left[g/mol\right]$$

(2) $${CO}_{2}\;Des=\left[\left(Q\times P\times \left(\left(C_{t}-0.04\;\%\right)/100\right)\times \left(10\;\left[s\right]/60[s/\min]\right)\times p_{{CO}_{2}}\right)-K_{loss}\right]/m_{AC}$$

The adsorption pressure in the AC bed is calculated according to Boyle's Law Equation (3) [34]. The main reason to determine this is that the outlet gas passes out through the flowmeter at a certain pressure; therefore, the outlet volume at ambient pressure needs to be known.

(3) $$P_{1}\times V_{1}=P_{2}\times V_{2}$$

**Fig. 2:**
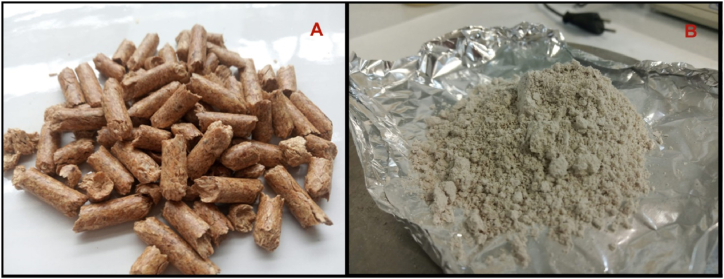
Pine pellets (A). Pine pellet ash (B).

The effect of the relative humidity on the adsorption capacity was studied by testing the adsorption capacity in triplicate at three inlet-gas humidity concentrations (3.5 %, 35 %, and 65 %). These relative humidity values were selected to evaluate the widest possible relative humidity range. To modify the relative humidity in the inlet gas, a moisturizing tube was filled with different water volumes to fix the inlet-gas relative humidity at the desired concentration; this was verified with the temperature/humidity sensor. To achieve the 3.5 % humidity concentration, the moisturizing tube remained empty due to the CO_2_ gas cylinder and the compressor almost having water content. Regarding the effect of the ash, four different concentrations (0 %, 0.01 %, 0.1 %, and 1 %) were evaluated. This ash concentration range was selected to determine this variable's effect on the CO_2_ adsorption capacity, considering a non-ash generation scenario up to a 1 % ash concentration, which is approximately what is expected when combusting pine pellets [19,35,36]. To study the ash effect, pine pellets were burnt in a furnace at 500 °C for 12 h. The ash (Fig. 2B) was collected and weighed to achieve the proportional mass studied in the AC bed. In the incoming gas stream, variable amounts of ash are injected through a premixing chamber. Regarding the inlet flow values (in litre per minute, LPM), these were selected to evaluate the variable's effect on the adsorption capacity. The inlet flow effect was studied by introducing different flows of CO_2_ gas and air; in total, four inlet flows were studied (1.2 LPM, 2.4 LPM, 3.6 LPM, and 4.8 LPM). Firstly, we considered the lowest inlet flow that could be achieved in the adsorption prototype where there would be no fluctuations in the rotameters and the CO_2_ and air flow in the inlet would be reliable. The lowest inlet flow selected was 1.2 LPM, and then three additional inlet flows were evaluated by increasing each one by a further 1.2 LPM compared to the previous inlet flow. All the tests were performed at a fixed CO_2_ concentration, adsorption pressure and inlet gas temperature of 8 %, 1.1 bar and 25 °C, respectively. An 8 % CO_2_ concentration was selected based on the expected CO_2_ concentration in biomass combustion gases [24,37]. This concentration is achieved by mixing CO_2_ with a purity about 99 % and outlet air. The necessary flow for introducing each gas was estimated using equation (4), where the CO_2_ concentration is the CO_2_ fraction in the gas mixture [%], Q_CO2_ is the CO_2_ flow introduced in the gas mixture [L/min], and Q_Air_ is the airflow introduced in the gas mixture [L/min].

(4) $${CO}_{2}\;Concentration=Q_{CO2}/\left(Q_{Air}+Q_{CO2}\right)$$

The desorption process is carried out by decreasing the pressure into the system, after that passing air free of additional CO_2_ through the bed till complete removal of CO_2_. Initially, the outlet valve is open to provide a constant gas flow from the bed, allowing the pressure of the activated carbon bed to steadily decrease. After reaching atmospheric pressure, ambient air is passed through the bed to favour the desorption process, the airflow is regulated to keep the pressure at a minimum value thus avoiding pressurizing the bed again. The airflow is maintained will the CO_2_ concentration at the outlet equals the atmospheric level.

### 2.3 Modelling

In line with previous works [[38], [39], [40], [41]], the data have been modelized according to the Langmuir isotherm **(5)**, Freundlich isotherm **(6)**, Tempkin isotherm **(7)** and Dubinin-Radushkevich isotherm **(8)**. These models were originally proposed to evaluate the effects of the pressure or the inlet gas CO_2_ concentration. However, in this work, we analysed the viability of these models for evaluating the effects of the inlet gas, relative humidity and ash content [42]. This was proposed taking into account the basic adsorption equation (10), where X is the CO_2_ adsorbed [g CO_2_/kg AC], P is the adsorption pressure [bar], [CO_2_] is the CO_2_ concentration at equilibrium, and K is a parameter. One would expect a higher humidity concentration in the gas to compete for the adsorption positions of the CO_2_ molecules, affecting the equivalent part of "log(P/[CO_2_])". In the case of the ash concentration, one would expect it to mainly affect the "K" factor. Nonetheless, to evaluate whether it could also compete with the adsorption positions of the CO_2_ molecules, the previously mentioned models were analysed. To study the viability of the Langmuir isotherm, the inverse of the studied variable data has been represented on the X-axis, and the inverse volume in cm^3^ of CO_2_ adsorbed/g AC has been represented on the Y-axis. Subsequently, the quadratic R-value was set to probe the linear correlation between this model and our data. The same validation was undertaken for the Freundlich isotherm but, in this case, the X-axis represents the logarithm of the studied variable data while the Y-axis represents the g CO_2_ adsorbed/kg AC [42], where V is the CO_2_ adsorbed [cm^3^ CO_2_/g AC], Vm is the monolayer volume [cm^3^], K is a nondimensional parameter, P is the adsorption pressure [bar], x is the CO_2_ adsorbed mass [g CO_2_], m is the AC mass [g AC], k is a parameter, and n is a parameter. In contrast, for the Tempkin model validation, the X-axis represents the typical data value while the Y-axis represents the exponential of the gr CO_2_ adsorbed/gr of AC, where K′1 and K2 are parameters. Finally, the Dubinin-Radushkevich model was evaluated by representing the result of equation (9) on the X-axis and the logarithm of the g CO_2_ adsorbed/kg AC on the Y-axis, where qs is the theoretical saturation capacity [mg CO_2_/kg AC], ε is the Polany potential [kJ/mol], and Ce is the CO_2_ concentration at equilibrium.

(5) (1/V)=(1/Vm)+[1/Vm×K]×(1/P)

(6) Log(x/m)=Log(k)+1/n×Log(P)

(7) $$\mathrm{EXP}\left({CO}_{2}\;Ads\right)=\mathrm{EXP}\left(K^{´}1\right)+K2\times Ce$$

(8) $$\mathrm{Log}\left({CO}_{2}\;Ads\right)=\mathrm{Log}\left(qs\right)-Kad\times \varepsilon^{2}$$

(9) $$\varepsilon^{2}=\left(RT\times \mathrm{Log}\left(1+1/Ce\right)\right){\;}^{2}$$

(10) $$X=K\times \mathrm{Log}(P/\left[{CO}_{2}\right]$$

Once the best model for each variable was analysed, the next step was to develop a theoretical equation to predict the adsorption capacity as a function of the variation in the variables within the range studied. To verify the reliability of this equation, the R^2^ value, Mean Bias Error (MBE) equation (11) and Mean Absolute Error (MAE) equation (12) were estimated from the correlation between the theoretical and experimental data [43].

(11) $$\text{MBE}=\left(1/n\right)\sum_{i=1}^{n}\left[\left(Theoretical\;data-Experimental\;data\right)/Experimental\;data\right]\times 100\%\;$$

(12) $$MAE=\left|\left(1/n\right)\sum_{i=1}^{n}\left[\left(Theoretical\;data-Experimental\;data\right)/Experimental\;data\right]\times 100\%\;\right|$$

### 2.4 Techno-economic analysis

In accordance with previous works [[44], [45], [46]], a techno-economic analysis was carried out following the CAPEX (Capital Expenditure) and OPEX (Operational Expenditure) methodologies. This analysis was undertaken to evaluate the economic viability of the adsorption process and thus the effect on the economic balance between the best and worst gas conditions assayed. In this work, a real case study has been considered - a 1000 m^2^ greenhouse equipped with a biomass boiler and a CO_2_ enrichment system using AC. In this system, the adsorption process is carried out each night between 20:00 and 08:00 when the greenhouse temperature drops and it is necessary to turn on the biomass boiler to heat the greenhouse. The AC bed has a capacity for 1000 kg of adsorbent material. During the daylight hours, between 08:00 and 20:00, the CO_2_ adsorbed in the AC bed is released into the greenhouse to enhance crop photosynthesis [24,47]. The 1000 kg of activated carbon costs 1000 € while the compressor, the AC bed and the instrumentation cost 2000 €, 1500 € and 3500 €, respectively. These data were obtained from AgroConnect (http://agroconnect.es/) [48]. The biomass cost is about 300 €/ton and the demand is approximately 287,437 kg/year with an electricity consumption of 15,813 kWh/year [48]. The proposed biomass is olive pits with a heating value of 20.7 MJ/kg [19], an ash content of 1.37 % [49], and a boiler efficiency of 80 % [50,51].

The economic balance of the adsorption process was estimated using equation (13), where the cost of energy in Spain is 0.15 €/kWh (June 2023) [52], the CO_2_ cost is 0.2 €/kg CO_2,_ and the cost of renting the CO_2_ tank is 3000 €/year [48]. Two scenarios were calculated to compare the difference between carrying out the adsorption at a relative gas humidity of around 65 % and an ash concentration of 1 % with that of a relative gas humidity of around 3 % and an ash concentration of 0 %. The comparison was performed using equation (13) where Balance is the profits per adsorption process [€/kg AC], P_CO2_ is the economic value of the CO_2_ saved in the adsorption process [€/s], and C_E_ is the electricity cost of pressurizing the activated carbon bed [€/s].

(13) $$Balance=\sum P_{CO2}-C_{E}$$

### 2.5 Activated carbon degradation

The objective of these tests was study whether the adsorption capacity of the “B-PURE 10-NB” AC from DESOTEC could be degraded due to poor conservation of the material. At the beginning, the active carbon was placed in four trays and stored correctly in its airtight bag meaning that the original material was sealed. Two of the trays were placed inside the laboratory, one sealed with transparent paper to allow the effects of temperature and radiation while avoiding humidity and particle deposition. The second tray was placed next to the first but was left uncovered. The third and fourth trays were prepared identically to the first and second, respectively, but were placed on the roof of the building, exposing them to direct radiation from the Sun and any atmospheric phenomena. All the tests were carried out in triplicate, taking AC samples from the original material and the four trays. First, the material was measured before placing it in its respective position and tray. Then, it was evaluated again two, four and six months later. The adsorption tests were carried out at a pressure of 1.1 bar and a CO_2_ concentration of 8 % following the same procedure discussed in the previous section.

## 3 Results and discussion

### 3.1 Influence of inlet flow on the adsorption capacity

**Fig. 3:**
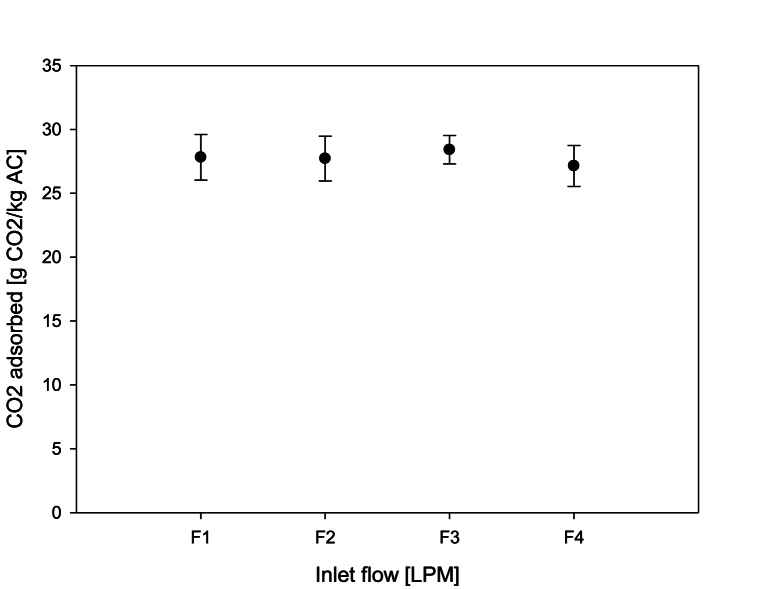
Effect on the adsorption capacity of different inlet flows at 8 % [CO_2_] and 1.1 bar of adsorption pressure.

**Fig. 4:**
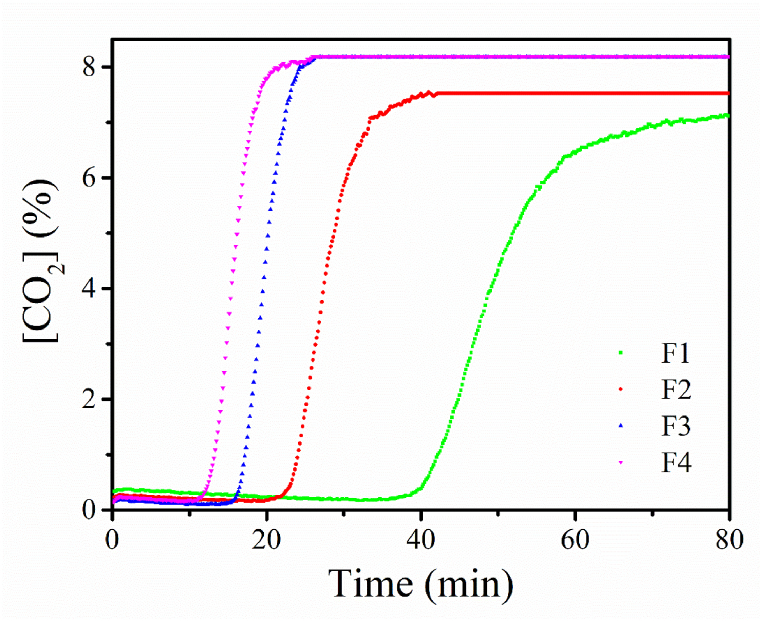
Effect of the inlet flow over the CO_2_ saturation curve, where F1 is 1.2 LPM, F2 is 2.4 LPM, F3 is 3.6 LPM and F4 is 4.8 LPM.

Different inlet flows can be set for the adsorption and desorption process. These tests aimed to determine if different inlet flows (at the same CO_2_ concentration and adsorption pressure) had any effect on the adsorption capacity of the AC. For all the inlet flows (Fig. 3) the adsorption capacity ranged between 26 and 29 g CO_2_/kg AC. No effect from the inlet flow was observed on the adsorption capacity within the studied range. However, different inlet flows showed a significant effect on the saturation curve times (Fig. 4) - an inlet flow of 1.2 LPM took 1 h and 19 min to saturate the AC used in the experiment whereas an inlet flow of 4.8 LPM only took 21 min to reach saturation. In other words, the saturation time was reduced by over 376 % from 1.2 LPM to 4.8 LPM without affecting the adsorption capacity. This data can be used to estimate the saturation times of different AC beds as a function of the inlet flow, CO_2_ concentration, and AC mass.

### 3.2 Influence of relative humidity on the adsorption capacity

**Fig. 5:**
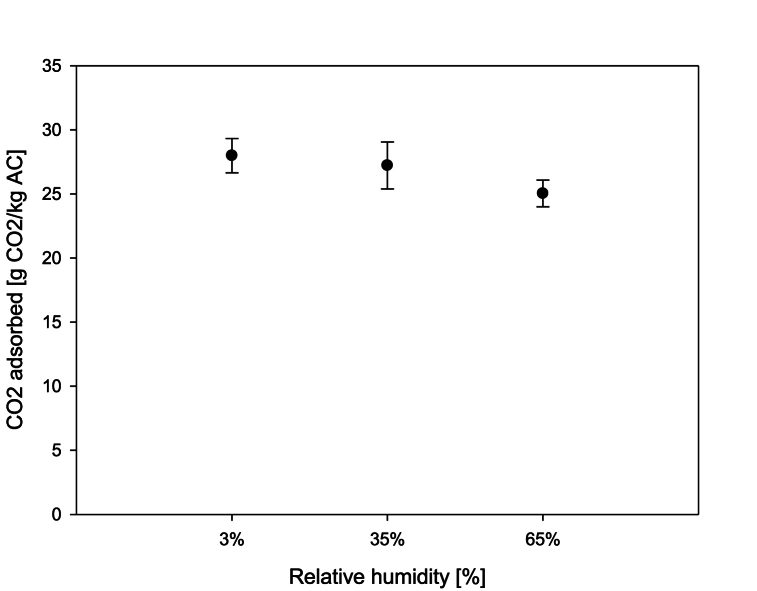
Effect on the adsorption capacity of different inlet flow relative humidities at 8 % [CO_2_] and 1.1 bar of adsorption pressure.

Biomass combustion is conditioned by the solid biofuel's characteristics. The relative humidities produced can range from 33 % in musasa (a dry African hardwood) to 220 % in fresh pine branches with needles, which negatively affects the adsorption capacity [53,54]. Zeolites are not a good choice for treating gases that have high relative humidities because they are much more sensitive to water and so quickly become saturated [55,56]. In these tests, the effect of the inlet gas relative humidity was evaluated at 3 %, 35 %, and 65 %. The results show that there is no difference in the adsorption capacity with relative humidities between 3 % and 35 % (Fig. 5). On the other hand, there is a 10.5 % decrease in the adsorption capacity between relative humidities of 3 % and 65 %. Nevertheless, the “B-PURE 10-NB” AC from DESOTEC presented a high humidity tolerance. These results are in line with those of other authors, demonstrating that there is a reduction in the adsorption capacity of the ACs even though the hydrophobic nature of most ACs reduces the negative effect of relative humidity. Water molecules are more effective in competing for adsorption centers on active carbons due to their polarity, smaller molecular size, the possibility of forming hydrogen bonds with functional groups on the carbon surface, and their greater chemical affinity with these centers, causing a decrease in the adsorption capacity, especially under conditions of high humidity, causing a reduction in the adsorption capacity [53,54,57]. In this work, the biomass was pine pellets, the moisture content of which is over 8 % of its mass [24], providing excellent conditions for the combustion process.

### 3.3 Influence of ash concentration on the adsorption capacity

**Fig. 6:**
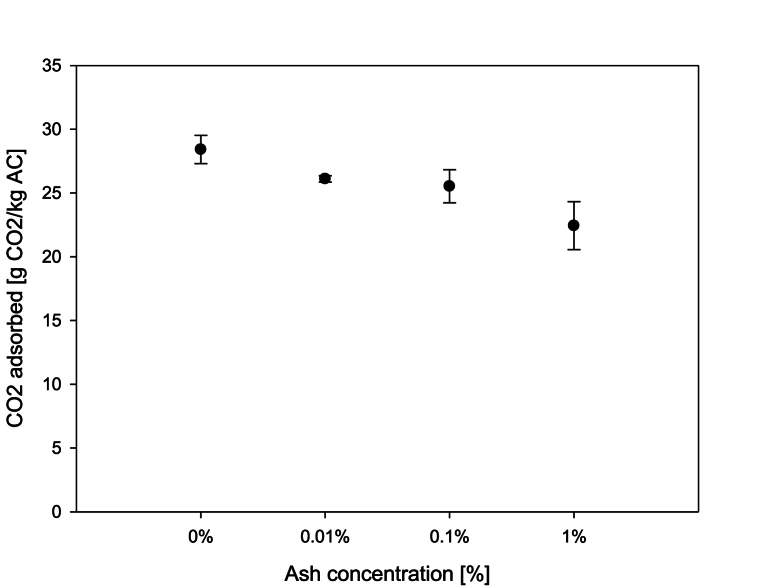
Effect on the adsorption capacity of an ash concentration at 8 % [CO_2_] and 1.1 bar of adsorption pressure.

Biomass combustion produces fly ash as a fraction of the mass burned. The fly ash produced varies as a function of the biomass burned, ranging from 0.37 % to 35.55 % [35,36,58]; for example, burning poplars and willows amounted to fly ash of 1.9 %–2 % whereas for the bark of coniferous trees, it was 3.9 % of the dry fuel mass [59]. The results (Fig. 6) show a negative effect on the adsorption capacity when comparing a clean adsorption process with no fly ash to an adsorption process with only 0.01 % ash with regard to the AC mass. There was no difference in the adsorption capacity between an ash concentration of 0.01 % and 0.1 % although the adsorption capacity was lower at an ash concentration of 1 % than at 0.01 %. In conclusion, the adsorption capacity decreased by 21 % over a range from 0 % to 1 % ash content with regard to the AC mass. This effect could be explained by the size and distribution of the micropores, mesopores and macropores since these determine the adsorptive properties of the ACs. If fly ash is deposited on the activated carbon, it might be blocking the macropore's access or even taking up the micropores and thus preventing the CO_2_ molecules from being adsorbed, resulting in less adsorption [60].

### 3.4 Modelling

**Fig. 7:**
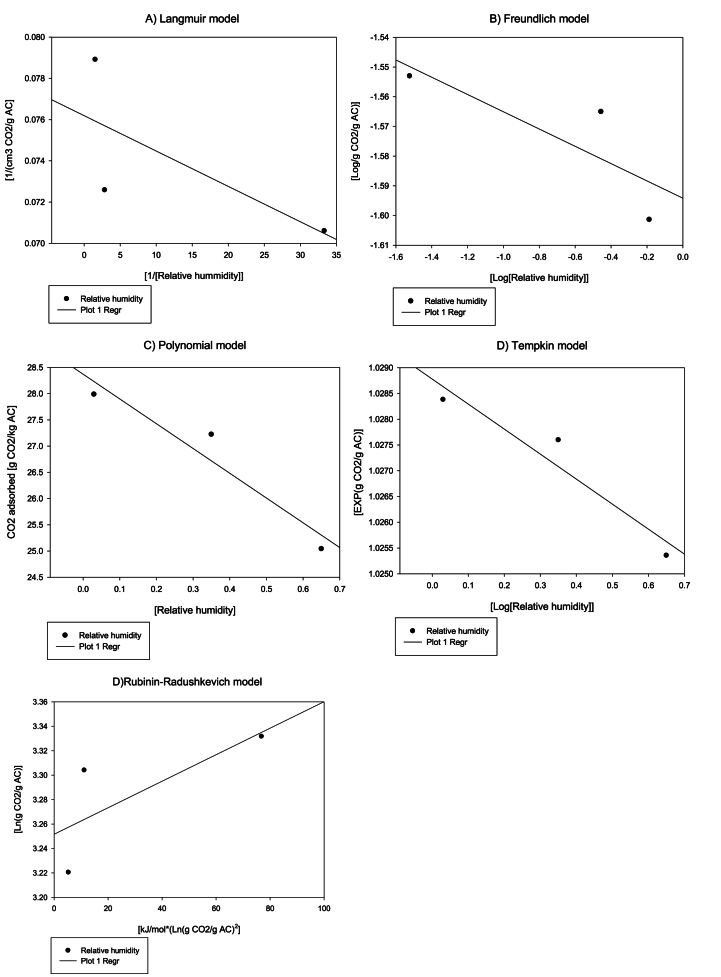
Inlet gas relative humidity effect over the CO_2_ adsorption capacity modelling, where A is the Langmuir model, B is the Freundlich model, C is a Polynomial model, D is the Tempkin model and D is the Rubinin-Radushkevich model.

**Fig. 8:**
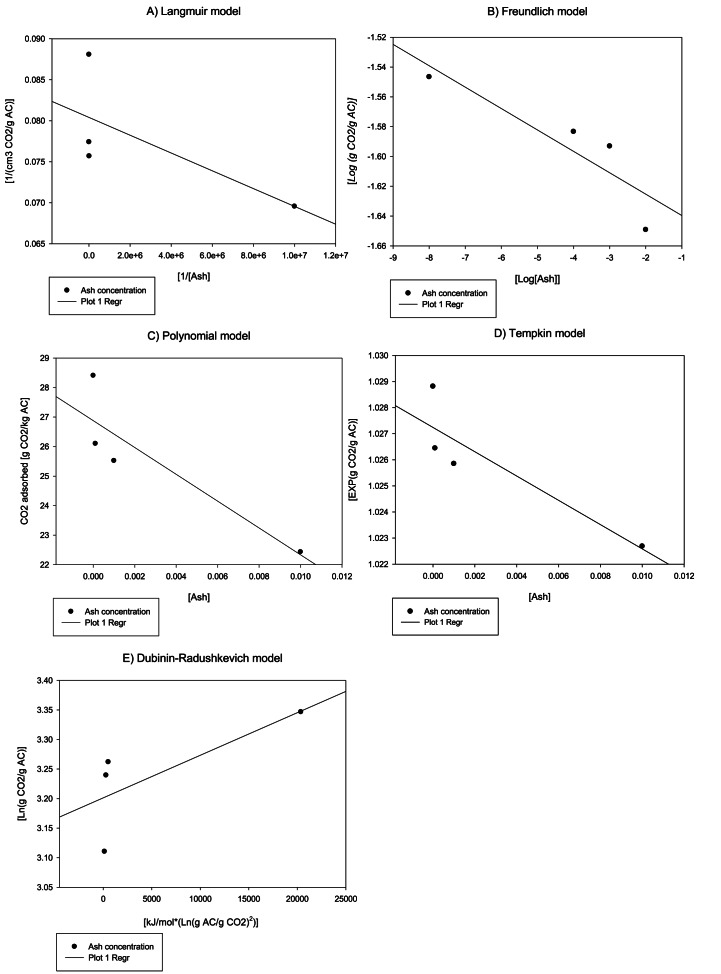
Ash concentration effect over the CO_2_ adsorption capacity modelling, where A is the Langmuir model, B is the Freundlich model, C is a Polynomial model, D is the Tempkin model and E is the Rubinin-Radushkevich model.

The influence of the two variables studied - inlet gas relative humidity and ash concentration - was modelled using the Langmuir, Freundlich, Tempkin and Dubinin-Radushkevich equations, thus as a polynomial equation (Fig. 7, Fig. 8) [42]. The inlet flow was not modelled as it had no any effect on the adsorption capacity. The inlet gas relative humidity data fitted with the Langmuir model, Freundlich model, Tempkin model, and Dubinin-Radushkevich with a reliability of 90.57 %, 66.74 %, 91.84 %, and 55.31 %, respectively. However, 100 % reliability was obtained for the Polynomial model (Fig. 7). The Langmuir and Tempkin models presented a fairly reliable R^2^ in terms of predicting the effect that this variable has on the material's adsorption capacity. As previously mentioned, its good fit is probably due to the water molecules competing with the CO_2_ molecules at the adsorption centers. The values obtained from the Tempkin model can be taken as being indicative of the expected adsorption depending on the inlet gas relative humidity. The ash concentration data on the other hand do not fit very well with any model, with the reliability of the Langmuir, Freundlich, Tempkin, and Dubinin-Radushkevich models and the Polynomial model being about 49.53 %, 79.29 %, 79.61 %, 54.68 %, and 80.1 %, respectively (Fig. 8). Consequently, the effect of the ash concentration cannot be modellized with significant reliability by any of these models. Therefore, the ash concentration effect would be exerted on parameter "K" in equation (10), almost certainly because of the obstruction of the porous structure, as previously mentioned.

In Table 2, the parameter values are compiled for the proposed models - the Langmuir, Freundlich, Tempkin, and Dubinin-Radushkevich - as the Polynomial and Exponential model for the inlet gas relative humidity and ash concentration, respectively. Based on the obtained data, equation (14) is proposed to estimate the adsorption capacity as a function of the relative humidity at an adsorption pressure of 1.1 bar and an inlet gas CO_2_ concentration of 8 %:

(14) $$\mathrm{EXP}\left({CO}_{2}\;\text{Ads}\right)=\mathrm{EXP}\left(1.014\right)-0.0049\times H$$

**Table 2:** Parameters for the Langmuir, Freundlich, Tempkin, and Dubinin-Radushkevich models as a function of the inlet gas relative humidity and ash concentration.

**Langmuir model**			

Inlet gas relative humidity	Vm	14.39	1.1 bar/8 % [CO_2_]
	K	5.23	1.1 bar/8 % [CO_2_]
Ash concentration	Vm	12.44	1.1 bar/8 % [CO_2_]
	K	−8.04E-9	1.1 bar/8 % [CO_2_]
**Freundlich model**			

Inlet gas relative humidity	k	0.03	1.1 bar/8 % [CO_2_]
	N	−34.36	1.1 bar/8 % [CO_2_]
Ash concentration	k	0.02	1.1 bar/8 % [CO_2_]
	N	−80.00	1.1 bar/8 % [CO_2_]
**Tempkin model**			

Inlet gas relative humidity	K2	−0.005	1.1 bar/8 % [CO_2_]
	K'1	0.028	1.1 bar/8 % [CO_2_]
Ash concentration	K2	−0.466	1.1 bar/8 % [CO_2_]
	K'1	0.027	1.1 bar/8 % [CO_2_]
**Dubinin-Radushkevich model**			

Inlet gas relative humidity	Kad	0.0011	1.1 bar/8 % [CO_2_]
	qs	35.83	1.1 bar/8 % [CO_2_]
Ash concentration	Kad	-7E-06	1.1 bar/8 % [CO_2_]
	qs	24.56	1.1 bar/8 % [CO_2_]

where CO_2_ ads. is the CO_2_ adsorbed in g CO_2_/kg AC and H is the inlet gas relative humidity as a decimal percentage. Table 2 shows the results of the statistical parameters estimated: the inlet gas relative humidity presented a reliability of 100 % while the MBE and MAE values were −0.0014 and 0.0014, respectively.

The four models evaluated are based on different physical assumptions about the adsorption process. The Langmuir model assumes monolayer adsorption on a homogeneous surface, and experimental data fits very well with this model because CO_2_ molecules tend to form a single layer on the surface until saturation is reached. The Tempkin model assumes that the heat of adsorption decreases linearly as the surface of the material becomes saturated due to adsorbate-adsorbate interactions, however, this makes it a more suitable model for intermediate adsorption phases. The Freundlich model describes the adsorption process on heterogeneous surfaces and allows the formation of multilayers, which is not the case for CO_2_ adsorption on activated carbon which is mainly performed as a single layer. On the other hand, the Dubinin-Radushkevich model focuses on physical adsorption in micropores, describing the relationship between the adsorbed amount and pressure through the characteristic energy of adsorption. The results obtained in this section coincide with the particularities of each model, with the Langmuir model being the most appropriate.

### 3.5 Economic analysis

The main aim of this paper is to optimize the adsorption process in order to generate the best possible yield. Three case studies were carried out. In the first case, the CO_2_ inlet gas concentration was 8 %, which is a typical value for biomass combustion [24,37]. We considered the standard operating conditions - a pressure of 1.1 bar at a temperature of 25 °C with an ash concentration of 0 % and an inlet gas relative humidity of 3 %. In the second case, we considered an adsorption pressure of 1.1 bar, an ash concentration of 1 %, and an inlet gas relative humidity of 65 %. This supposes CO_2_ adsorption values of 28 g CO_2_/kg AC and 16 g CO_2_/kg AC for the first and second case, respectively. The third case study used liquified CO_2_ instead of the proposed adsorption system, a scenario in which all the CO_2_ that is expected to be adsorbed by the AC comes from liquified CO_2_. The adsorption process operates 365 days a year at complete AC bed saturation. The AC bed has a capacity of 1000 kg of AC, thus the maximum CO_2_ adsorbed is expected to be around 10,220 kg CO_2_/year under the best conditions and 5840 kg CO_2_/year under the worst. The annual biomass consumption considered is 287,437 kg to provide sufficient energy to heat the greenhouse throughout the year. This consumption involves CO_2_ emissions of 16,757 kg CO_2_/year. In such a situation, the AC bed is unable to adsorb all the CO_2_ produced from the biomass boiler combustion under the worst or best conditions because the maximum adsorbed per day under the best condition is only 10,220 kg CO_2_. In this scenario, the AC bed needs to have a capacity of 2870 kg to be able to adsorb all the CO_2_ produced by biomass combustion under the worst conditions and 1640 kg under the best, thus increasing the activated carbon cost.

The CAPEX and OPEX analyses are set out in Table 3. They consider the costs of a kg of CO_2_ for the three scenarios studied. The total production costs per year under the worst combustion gas conditions, and when using liquified CO_2,_ are 2835 €/year. In the two adsorption scenarios, the CO_2_ adsorbed in the AC bed is used to enhance the photosynthesis process in the greenhouse instead of using liquified CO_2_. This CO_2_ utilization represents a saving of 0.25 €/kg CO_2_ under the optimal conditions and 0.17 €/kg under the worst conditions assayed. Moreover, the adsorption process avoids 16,757 kg of CO_2_ emissions per year. However, when comparing the best and worst operating conditions, a saving of only 0.08 €/kg CO_2_ is expected. The total savings from the best adsorption conditions compared to using liquified CO_2_ is around 60 % whereas between the best and worst operating conditions, it is around 12 %.

**Table 3:** CAPEX and OPEX analysis for a combined system of heating and carbon enrichment from biomass.

TOTAL COSTS		LIQUIFIED CO_2_	
Item	Details	Cost, €	Percentage
1	Total fixed capital per annum	0.00 €	0.0 %
2	Total raw materials	6351.52	85.7 %
3	Total utilities	–	0.0 %
4	Total labour and others	1063.23	14.3 %
**Total fixed capital per annum**			**0.00**
**Total direct production costs**			**7414.75**
**Total production costs (€)**			**7414.75**
**Unit cost of adsorbed CO**_**2**_**(€/kg)**			**0.44**

TOTAL COSTS		ADSORPTION - OPTIMAL CONDITIONS	
Item	Details	Cost, €	Percentage
1	Total fixed capital per annum	1984.23 €	61.8 %
2	Total raw materials	–	0.0 %
3	Total utilities	163.94	5.1 %
4	Total labour and others	1063.23	33.1 %
**Total fixed capital per annum**			**1984.23**
**Total direct production costs**			**1227.18**
**Total production costs (€)**			**3211.41**
**Unit cost of adsorbed CO**_**2**_**(€/kg)**			**0.19**

TOTAL COSTS		ADSORPTION - WORST CONDITIONS	
Item	Details	Cost, €	Percentage
1	Total fixed capital per annum	3188.78 €	69.6 %
2	Total raw materials	–	0.0 %
3	Total utilities	327.89	7.2 %
4	Total labour and others	1063.23	23.2 %
**Total fixed capital per annum**			**3188.78**
**Total direct production costs**			**1391.12**
**Total production costs (€)**			**4579.90**
**Unit cost of adsorbed CO**_**2**_**(€/kg)**			**0.27**

### 3.6 Activated carbon degradation

**Fig. 9:**
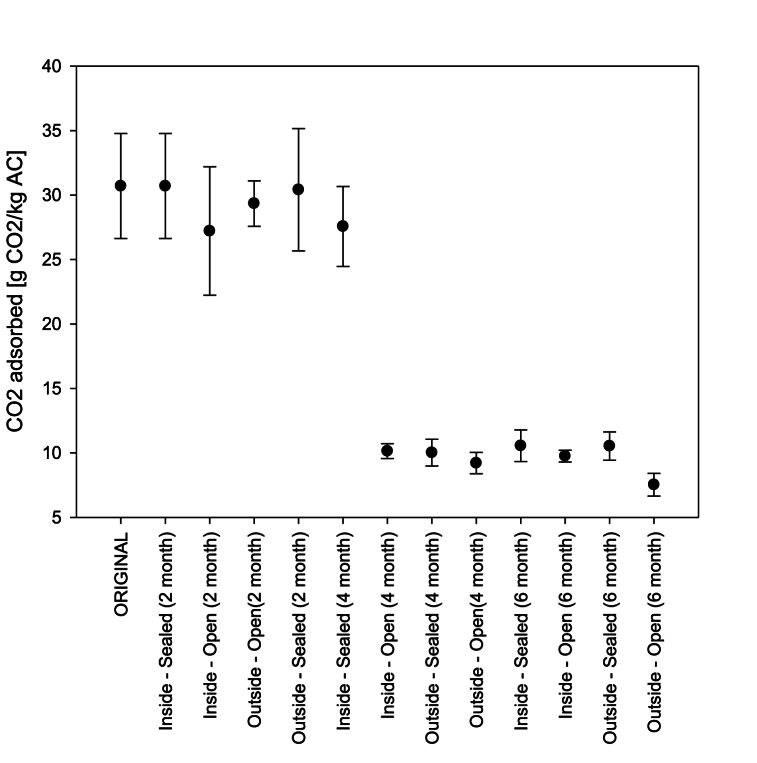
Activated carbon degradation evaluation, where X-axis show the time and material conservation carried out.

The capacity of CO_2_ adsorption of the activated carbon stored at different conditions was studied, the storage conditions being defined to mimic that potentially found in the use of the proposed process in greenhouses. Results from these experiments are shown compiled in Fig. 9. After two months, none of the samples taken from the four trays showed significant variations concerning the original material. After four months, only the sealed material tray inside the laboratory still had an adsorption capacity of 30 g CO_2_/kg AC, whereas the other samples saw their capacity drop to 10 g CO_2_/kg AC. This effect was due to the accumulation of suspended particles deposited on the material in the open trays and greater ambient humidity captured by the sealed material tray exposed to the outside environment. Finally, after six months of exposure, all the materials placed on the trays had their adsorption capacity reduced to 10 g CO_2_/kg AC, except for the open tray located outside, for which the capacity dropped to around 7 g CO_2_/kg AC. This was a consequence of a greater number amount of fine particles clogging the material's porous structure since significant dust accumulation was visible in the tray. Good material storage practices are necessary to avoid a loss in adsorption capacity, both for the material stored for later use and for the material placed in the adsorption tank, minimising exposure to dust and isolating it from outside humidity. When introducing a new material into an adsorption tank, prior thermal desorption at 70-100 °C for 24 h is recommended to eliminate the moisture retained by the material.

## 4 Conclusions

The inlet flow rate was found to have no effect on the adsorption capacity although a higher inlet flow reduces the time in which the saturation curve is reached. On the other hand, higher inlet gas relative humidities of between 3 % and 65 % reduce the adsorption capacity by 10.5 %. A higher ash concentration in the 0 %–1 % range also reduces the adsorption capacity by 21 %. Neither the Langmuir, Freundlich, Tempkin, or Dubinin-Radushkevich's models presented a good fit with the data for the inlet gas relative humidity or ash concentration. However, a polynomial equation did fit the data with 100 % reliability. The economic analysis performed considered a 1000 m^2^ greenhouse. The comparable savings obtained in adsorbed CO_2_ between the best and worst operating conditions were around 12 % and 60 %, respectively, compared to using liquified CO_2_. In this work, the CO_2_ adsorption process was developed with the idea of reducing the CO_2_ emissions from the hybrid system and obtaining extra profit by reusing the adsorbed CO_2_. Considering that the profits generated come from CO_2_ savings from biomass boiler combustion, the combined heating and carbon enrichment system is expected to be profitable. Furthermore, good conservation of the adsorbent material is essential, otherwise around 75 % of its adsorption capacity could be lost.

## CRediT authorship contribution statement

**R. López Pastor:** Writing – original draft, Methodology, Investigation, Formal analysis, Conceptualization. **M.G. Pinna-Hernández:** Investigation, Formal analysis, Writing – review & editing, Supervision. **J.A. Sánchez Molina:** Investigation, Formal analysis, Writing – review & editing, Supervision. **F.G. Acién Fernández:** Investigation, Formal analysis, Writing – review & editing, Supervision.

## Declaration of competing interest

The authors declare that they have no known competing financial interests or personal relationships that could have appeared to influence the work reported in this paper.
